# Adipose-derived exosomes protect the pulmonary endothelial barrier in ventilator-induced lung injury by inhibiting the TRPV4/Ca^2+^ signaling pathway

**DOI:** 10.1152/ajplung.00255.2019

**Published:** 2020-02-19

**Authors:** Qian Yu, Daoxin Wang, Xiaoting Wen, Xumao Tang, Di Qi, Jing He, Yan Zhao, Wang Deng, Tao Zhu

**Affiliations:** Department of Respiratory Medicine, Second Affiliated Hospital of Chongqing Medical University, Chongqing, China

**Keywords:** adipose-derived exosomes, inflammation, pulmonary endothelial barrier, TRPV4, ventilator-induced lung injury (VILI)

## Abstract

Mechanical ventilation (MV) is the main supportive treatment of acute respiratory distress syndrome (ARDS), but it may lead to ventilator-induced lung injury (VILI). Large epidemiological studies have found that obesity was associated with lower mortality in mechanically ventilated patients with acute lung injury, which is known as “obesity paradox.” However, the effects of obesity on VILI are unknown. In the present study, wild-type mice were fed a high-fat diet (HFD) and ventilated with high tidal volume to investigate the effects of obesity on VILI in vivo, and pulmonary microvascular endothelial cells (PMVECs) were subjected to 18% cyclic stretching (CS) to further investigate its underlying mechanism in vitro. We found that HFD protects mice from VILI by alleviating the pulmonary endothelial barrier injury and inflammatory responses in mice. Adipose-derived exosomes can regulate distant tissues as novel adipokines, providing a new mechanism for cell-cell interactions. We extracted three adipose-derived exosomes, including HFD mouse serum exosome (S-Exo), adipose tissue exosome (AT-Exo), and adipose-derived stem cell exosome (ADSC-Exo), and further explored their effects on MV or 18% CS-induced VILI in vivo and in vitro. Administration of three exosomes protected against VILI by suppressing pulmonary endothelial barrier hyperpermeability, repairing the expression of adherens junctions, and alleviating inflammatory response in vivo and in vitro, accompanied by transient receptor potential vanilloid 4 (TRPV4)/Ca^2+^ pathway inhibition. Collectively, these data indicated that HFD-induced obesity plays a protective role in VILI by alleviating the pulmonary endothelial barrier injury and inflammatory response via adipose-derived exosomes, at least partially, through inhibiting the TRPV4/Ca^2+^ pathway.

## INTRODUCTION

Although acute respiratory distress syndrome (ARDS)-related research has been ongoing for more than 40 years, mortality due to the disease is still high, and there are no effective treatment measures. Mechanical ventilation (MV) is the main supportive treatment, but it may lead to ventilator-induced lung injury (VILI) and cause further pulmonary vascular permeability increases, pulmonary edema, and inflammatory cell infiltration (45). At present, the methods used to reduce VILI mainly include low-tidal mechanical ventilation, the use of the prone position, high-frequency vibration, high positive end-expiratory pressure (PEEP), and others (16), but reductions in ARDS-related mortality remain elusive.

Currently, relevant studies have found that obese patients have a higher risk of ARDS than normal weight patients. However, those who were obese had higher survival rates (41). Obesity and overweight are often associated with an increased risk of death in the general population, but in some critical diseases such as ARDS, mortality is reduced, which is known as the “obesity paradox” (3, 31). The same phenomenon has been shown in patients undergoing mechanical ventilation (2, 39, 58). A recent animal study found that a high-fat diet (HFD) can protect mice from VILI via neutrophil-independent mechanisms (51), driving us to further explore the protective effects and mechanisms of obesity on VILI.

Why does obesity play a protective role in ARDS and VILI? Adipose tissue is regarded as an important endocrine organ that participates in the regulation of physiological functions by secreting a variety of adipocytokines. For example, omentin can alleviate structural and functional damage of pulmonary microvascular endothelial cells (PMVECs) (36), leptin levels were higher in obese patients and were positively correlated with survival in sepsis patients (32), and interleukin-10 (IL-10) secreted by adipocytes can inhibit the inflammatory response (1). Adipose-derived exosomes are genetic adipokines secreted by adipose tissue and can act on distant tissues, providing a new mechanism for cell-cell interactions (43). As extracellular vesicles with diameters of 30–150 nm, exosomes can regulate the biological activities of recipient cells by transporting proteins, nucleic acids, and lipids (17). Main sources of adipose-derived exosomes in previous researches included adipose tissue explants, mouse serum, rat primary adipocytes, and differentiated 3T3-L1 cell lines (11). Exosomes derived from adipose-derived stem cells (ADSCs) show protective effects on many diseases, which can attenuate the inflammatory response (57), improve vascular formation, and promote wound healing (5, 27).

Transient receptor potential vanilloid 4 (TRPV4) in membrane ion channels has been found to mediate pulmonary vascular permeability induced by high-pressure mechanical ventilation (23). As a mechanically sensitive receptor, TRPV4 is highly expressed in the pulmonary endothelium, and the activation of TRPV4 leads to increased intracellular Ca^2+^, endothelial cell barrier destruction, and pulmonary edema (49). Selective inhibition of TRPV4 can reduce the pulmonary barrier hyperpermeability and the proinflammatory cytokines release (34).

Therefore, our study aims to verify the effects of adipose-derived exosomes (from serum, adipose tissue, and ADSC) on inflammation and the endothelial barrier in VILI. Furthermore, we mechanistically investigated the role of TRPV4/Ca^2+^ signaling pathway in these effects induced by adipose-derived exosomes in vivo and in vitro.

## MATERIALS AND METHODS

### 

#### VILI animal model and treatment of exosomes.

Starting at 4 wk, male SPF C57BL/6 mice were fed an HFD (45% of total calories) for 3 mo, whereas others were fed a normal chow diet (NCD) for 3 mo. Mice were anesthetized with pentobarbital sodium (50 mg/kg intraperitoneally), and MV was applied (ALC-V8S small animal ventilator; Shanghai Alcott Biotechnology Co., Ltd., Shanghai, China) at a tidal volume (Vt) of 30 mL/kg, a respiratory rate of 75 breaths/min, and a PEEP of 0 cmH_2_O for 4 h (in vivo model of VILI) (37). The main reasons why we chose 30 mL/kg as the parameter of VILI, much higher than clinical ventilation regime, are as follows. First, mice have highly compliant rib cages and abdominal walls, and mouse lungs are much more compliant than human lungs (61); therefore, such high Vt is necessary to induce lung injury within healthy animals (50). Second, we matched the similar respiratory mechanics induced by ventilation between lean and fat-fed obese mice, ensuring that the lungs are effectively exposed to the same degree of resultant stretch (in terms of same Vt) (51). Third, the safety and efficiency of ventilation strategy was confirmed by preliminary experiments in which arterial blood gases analysis revealed stable levels of arterial oxygen ($\text{P}{\text{a}}_{\text{O}}{}_{{}_{2}}$ of 55–95 mmHg) and carbon dioxide ($\text{P}{\text{a}}_{\text{C}\text{O}}{}_{{}_{2}}$ of 35–50 mmHg). Three types of exosomes, namely, HFD mice serum exosome (S-Exo), adipose tissue (AT) exosome (AT-Exo), and ADSC exosome (ADSC-Exo), were intravenously administered at different concentrations (0, 25, 50, and 100 μg/mL) in a total volume of 200 μL of phosphate-buffered saline (PBS) 1 h before MV to determine the optimal exosome intervention concentration. Exosomes were diluted with sterile PBS, bathed at 37°C, and injected slowly and uniformly for >5 min. The groups included the NCD + spontaneously breathing (SB) group, NCD + MV group, HFD + SB group, HFD + MV group, MV + S-Exo/AT-Exo/ADSC-Exo (0, 25, 50, and 100 μg/mL) group, SB group, MV group, MV + PBS (200 μL) group, MV + exosome-depleted HFD mouse serum (Exo-depleted-Serum)/exosome-depleted AT conditioned media (Exo-depleted-AT-CM)/exosome-depleted ADSC conditioned media (Exo-depleted-ADSC-CM) (at the same concentrations as the corresponding exosomes) group, and MV + S-Exo/AT-Exo/ADSC-Exo group. Furthermore, mice were treated with the combination of ADSC-Exo and TRPV4 agonist GSK1016790A (6 nM; MedChemExpress) or TRPV4 antagonist HC-067047 (20 nM; MedChemExpress) at the onset of MV (29) to confirm the involvement of TRPV4 signaling in adipose-derived exosomes-mediated protection against VILI in vivo. To explore the therapeutic effects of ADSC-Exo on the VILI mouse model, ADSC-Exo was intravenously administered immediately after MV, and mice were euthanized 4 h after ADSC-Exo administration.

#### Isolation and identification of ADSC.

All animal experimental protocols were conducted according to the guidelines of the National Institutes of Health’s *Guide for the Care and Use of Laboratory Animals* and were approved by the Ethics Committee of Chongqing Medical University. The inguinal adipose tissue of 4-wk-old SPF male C57BL/6 mice was washed three times with ice-cold PBS containing 100 U/mL penicillin-streptomycin liquid (Solarbio Technology Co., Ltd., Beijing, China) to remove blood vessels, fat fascia, and connective tissue. The adipose tissue was cut into pieces of ∼1 × 1 × 1 mm, digested in 0.2% of type I collagenase (Sigma) in a 37°C water bath for 30 min with shaking every 5 min, and centrifuged at 1,000 rpm/min for 5 min after 30 min. An equal amount of DMEM-F-12 (Thermo Scientific HyClone) culture medium containing 15% fetal bovine serum (Lonsera) and 1% penicillin-streptomycin liquid was added to stop digestion, and the tissue was filtered through a 200-mesh cell screen. The filtrate was centrifuged at 1,000 rpm/min for 10 min, and the supernatant was discarded. The pellet was separated into a cell suspension in DMEM-F-12 complete medium and inoculated into a 25-cm^2^ culture flask that was incubated at 37°C in 5% CO_2_ and 95% humidity in a culture incubation box. The culture medium was changed for the first time after 48 h and then was changed every 2 days. When the cells were 80–90% confluent, they were detached with 0.25% trypsin and subcultured at a ratio of 1:3. For the flow cytometry detection of ADSC immunophenotypes, third-generation ADSCs were used. When the cells had grown to 80–90% confluence, they were trypsinized, centrifuged, and washed in PBS twice, and ∼10^6^ cells/tube were resuspended in 100 μL of PBS. APC anti-mouse CD45 antibody (BioLegend, Inc.), FITC anti-mouse CD31 antibody (BioLegend, Inc.), and APC anti-mouse/human CD44 antibody (BioLegend, Inc.) were added, and the solution was incubated at 4°C for 30 min, after which the unlabeled antibody was washed away with PBS, and the cells were resuspended in 200 μL of PBS. The isotype antibody served as a control. The cell phenotypes were detected by CytoFLEX (Beckman Coulter, Inc.) flow cytometry, and the results were analyzed using CytExpert software. For the identification of the adipogenic differentiation of ADSCs, third-generation ADSCs were inoculated into a six-well plate and grown to 80–90% confluence, and adipogenic induction solution [DMEM-F-12, 100 U/mL penicillin, 100 U/mL streptomycin, 0.25 μmol/L dexamethasone (Sigma), 10 mg/L insulin (Solarbio Technology Co., Ltd., Beijing, China), 0.2 mmol/L indomethacin (Solarbio Technology Co., Ltd., Beijing, China), and 0.5 mmol/L 3-isobutyl-1-methylxanthine (Sigma)] was added. Two weeks after induction, two-thirds of the culture medium was changed every 2 days. After induction, the cells were washed with PBS, fixed for 30 min in 4% paraformaldehyde (Solarbio Technology Co., Ltd., Beijing, China), stained with Oil Red O stain for 20 min (Solarbio Technology Co., Ltd., Beijing, China) and hematoxylin for 2 min (Solarbio Technology Co., Ltd., Beijing, China), and washed with PBS. The lipoblast staining effect was observed under a microscope. Oil Red O-positive cells were counted in three randomly selected fields of each slide by a blinded investigator.

#### Extraction and identification of exosomes.

Serum exosomes were collected using Total Exosome Isolation (from serum) reagent (Invitrogen). First, the serum sample was centrifuged at 2,000 *g* for 30 min to remove cells and debris. Then, the required volume of clarified serum was transferred to a new tube, and 0.2 volumes of Total Exosome Isolation reagent was added. The serum-reagent mixture was mixed well by vortexing until there was a homogenous solution. Next, the sample was incubated at 4°C for 30 min. After incubation, the sample was centrifuged at 10,000 g for 10 min at room temperature. Finally, the supernatant was discarded, and the exosomes that were contained in the pellet at the bottom of the tube were resuspended in PBS buffer. The extraction of adipose tissue exosomes was also performed. Fetal bovine serum was centrifuged at 100,000 *g* for 12 h, and the supernatant was used to prepare the exosome-free fetal bovine serum. The visceral adipose tissue of C57BL/6 male mice was washed with cold PBS to remove connective tissue and blood clots, cut into <4-mm pieces, and transferred to a six-well plate containing 10% exosome-free fetal bovine serum and 50 μg/mL gentamicin in DMEM (Invitrogen), which was incubated at 37°C in 5% CO_2_. The culture supernatant was used for exosome purification by differential centrifugation 48 h later (15). For the extraction of ADSC exosomes, third-generation ADSCs were used. When the cells were cultured to 80–90% confluence, the medium was replaced with 10% exosome-free fetal bovine serum. After 48 h of culture, the collected culture supernatant was centrifuged at 4°C and 300 *g* for 10 min, centrifuged at 2,000 *g* for 10 min to remove cell debris, centrifuged at 10,000 *g* for 30 min to remove larger cell vesicles, and centrifuged at 100,000 *g* for 70 min (Hitachi High-Technologies Corporation CP100MX, Hitachi, Ltd., Tokyo, Japan). The supernatant was discarded, and the pellet was resuspended in precooled PBS. The exosomes were washed again by centrifugation at 100,000 *g* for 70 min, and the supernatant was discarded. The exosomes were resuspended in a volume of 200 μl of precooled PBS (42) and stored at −80°C. For detection by electron microscopy, a transmission electron microscope (TEM; HITACHI HT7700 TEM, Hitachi, Ltd., Tokyo, Japan) was used for observation and image analysis. For nanoparticle tracking analysis (NTA), an NTA system from Malvern (Nano ZS ZEN3600, Malvern Panalytical Ltd., Worcestershire, UK) was used to measure the sample size distribution. For Western blot analysis, the protein expression levels of the representative exosome markers CD63 (1:1,000; Proteintech Group Inc., Chicago, IL), HSP70 (1:2,000; Proteintech Group Inc., Chicago, IL), and TSG101 (1:1,000; Proteintech Group Inc., Chicago, IL) were detected.

#### Lung histopathology.

After the experiment, the lungs were dissected and fixed immediately in 4% paraformaldehyde, embedded in paraffin, cut into 4-μm sections, and stained with hematoxylin and eosin (H & E). Three slides from each mouse were evaluated for a total of four mice per group. The degree of lung injury was evaluated by a semiquantitative scoring system, as previously described (48).

#### Lung wet/dry weight ratio.

The right lung tissue was immediately removed after the experiment, weighed, and then dried in an oven at 55°C for 72 h. Finally, the dry weight was measured, and the wet/dry (W/D) ratio was calculated.

#### Protein concentration in bronchoalveolar lavage fluid.

After the experiment, alveolar lavage was performed by infusing 1 mL of normal saline into the lungs through tracheal intubation, and ∼0.8 mL of bronchoalveolar lavage fluid (BALF) was recovered and centrifuged at 500 *g* for 20 min at 4°C. The protein concentrations of the BALF supernatants were determined using a BCA Protein Assay Kit (Solarbio Technology Co., Ltd., Beijing, China).

#### Myeloperoxidase assay.

The concentration of myeloperoxidase (MPO) in lung tissue, an index of neutrophil sequestration, was measured using the Myeloperoxidase Assay Kit (Nanjing Jiancheng Bioengineering Institute, Nanjing, China).

#### Cytokine assay.

The levels of the tumor necrosis factor-α (TNF-α) and interleukin (IL)-6 cytokines in the cell culture supernatant and BALF supernatant were assayed using commercial ELISA kits according to the manufacturer’s instructions (Cloud-Clone Corp, Wuhan, China).

#### The 18% cyclic stretching of cells and treatment of exosomes.

To determine the effects of exosomes on murine PMVECs under cyclic stretching (CS), PMVECs were purchased from Procell Life Science & Technology Co., Ltd. (Wuhan, China) and cultured in the complete medium recommended by the manufacturer for PMVECs, which contained 10% fetal bovine serum. Cells were grown at 37°C in a 5% CO_2_ incubator and used at passages 2–5. PMVECs were plated at 4 × 10^5^ cells/well in Flexcell biaxial six-well plates (coated with type I collagen, BioFlex-I; Flexcell International Corp.) overnight. The culture medium was replaced 24 h before the cells were subjected to 18% CS for 4 h at 0.5 Hz (in vitro model of VILI) and 5% CS for 4 h at 0.5 Hz (in vitro model of SB) using a Flexcell FX-4000 Tension System (Flexcell International Corp.). We added S-Exo, AT-Exo, or ADSC-Exo at different concentrations (0, 25, 50, and 100 μg/mL) 1 h before 18% CS to determine the best exosome intervention concentration. The groups included the 18% CS + S-Exo/AT-Exo/ADSC-Exo (0, 25, 50, and 100 μg/mL) group, 5% CS group, 18% CS group, 18% CS + PBS (with the same volume as the corresponding exosomes) group, 18% CS + Exo-depleted-Serum/Exo-depleted-AT-CM/Exo-depleted-ADSC-CM (with the same concentration as the corresponding exosomes) group, and 18% CS + S-Exo/AT-Exo/ADSC-Exo group. Cells were treated with the combination of ADSC-Exo and TRPV4 agonist GSK1016790A (300 nM) or TRPV4 antagonist HC-067047 (1 μM) at the onset of 18% CS (30, 46) to confirm the involvement of TRPV4 signaling in adipose-derived exosomes-mediated protection against VILI in vitro. To explore the therapeutic effects of ADSC-Exo on the VILI model in vitro, ADSC-Exo was administered immediately after 18% CS, and then the PMVECs and its culture supernatant were collected 4 h after ADSC-Exo administration.

#### Uptake of exosomes by PMVECs and lung tissue.

PMVECs were seeded in a six-well plate. Cells were treated with PKH26 (Sigma)-labeled S-Exo/AT-Exo/ADSC-Exo for 1 h. PKH26-labeled S-Exo/AT-Exo/ADSC-Exo was intravenously administered to wild-type mice. After 1 h, the mice were euthanized. Immunofluorescent staining was performed in cells and lung sections. The nuclei were stained with DAPI (Sigma), the vascular endothelial cells were stained with FITC-labeled CD31 (Cell Signaling Technology), and the cells with red fluorescence indicated the uptake of PKH26-labeled Exo. For each group, the infusion of PBS stained with PKH26 was used as a control. We observed the cells and lung tissue using fluorescence microscope (BX51 Olympus, Tokyo, Japan).

#### Quantitative real-time PCR.

The levels of TNF-α, IL-6, TRPV4, β-catenin, VE-cadherin, and glyceraldehyde-3-phosphate dehydrogenase (GAPDH) mRNA expression were analyzed by RT-PCR. Total RNA was extracted from PMVECs or lung tissue using RNAiso Plus (Takara Biomedical Technology Co., Ltd., Beijing, China). RNA samples were quantified using a NanoPhotometer N50 (Implen, München, Germany). Next, 1 μg of total RNA was used for amplifying the cDNA using the PrimeScript RT reagent kit with gDNA Eraser (Takara Biomedical Technology Co., Ltd., Beijing, China). Then, 2 µL of cDNA was used for qPCR amplification in a 25-µL reaction mixture containing 12.5 µl of TB Green Premix Ex *Taq* II (Tli RNaseH Plus; Takara Biomedical Technology Co., Ltd., Beijing, China). The specific primer sequences (Takara Biomedical Technology Co., Ltd., Beijing, China) used for the quantitative real-time PCR (qRT-PCR) assays are as follows: TNF-α (sense: 5′-GCACCACCATCAAGGACTCAA-3′, antisense: 5′-CAGGGAAGAATCTGGAAAGGTC-3′); IL-6 (sense: 5′-CTTGGGACTGATGCTGGTGAC-3′, antisense: 5′-TTCTCATTTCCACGATTTCCCA-3′); TRPV4 (sense: 5′- CGAGCGTTCCTTCCCTGTGTT-3′, antisense: 5′-AGACCAGTTCACCTCGTCCACC-3′); β-catenin (sense: 5′-GTTCTACGCCATCACGACACTG-3′, antisense: 5′-TTGCTCTCTTGATTGCCATAAGC-3′); VE-cadherin (sense: 5′-CCAGCCCTACGAACCTAAAGTG-3′, antisense: 5′-GGTTTACTGGCACCACATCCTT-3′); and GAPDH (sense: 5′-GACATCAAGAAGGTGGTGAAGC-3′, antisense: 5′-GAAGGTGGAAGAGTGGGAGTT-3′). GAPDH was used as the endogenous control gene. The relative mRNA expression was determined using the comparative C_T_ method ($2^{{}^{–\Delta \Delta {\text{C}}_{\text{T}}}}$).

#### Western blot analysis.

To determine protein expression levels, whole cell or lung tissue extracts were lysed with RIPA buffer (Solarbio Technology Co., Ltd., Beijing, China) containing 1% phenylmethylsulfonyl fluoride (PMSF). The protein concentration was determined using the BCA Protein Assay Kit (Solarbio Technology Co., Ltd., Beijing, China). Then, the protein was resolved by electrophoresis on an SDS polyacrylamide gel and electrophoretically transblotted onto polyvinylidene fluoride (PVDF) membranes. The PVDF membranes were incubated in blocking solution for 1 h and then probed with primary and secondary antibodies. The primary antibodies used in this work are as follows: TNF-α (1:1,000, Proteintech Group Inc., Chicago, IL), IL-6 (1:1,000, Proteintech Group Inc., Chicago, IL), TRPV4 (1:2,000, GeneTex, Inc.), β-catenin (1:3,000, Proteintech Group Inc., Chicago, IL), VE-cadherin (1:2,000, Abcam), and GAPDH (1:3,000, Proteintech Group Inc., Chicago, IL). The appropriate secondary antibody, either goat anti-mouse IgG (H + L)-HRP (1:6,000, Bioworld Technology, Inc.) or goat anti-rabbit IgG (H + L)-HRP (1:6,000, Bioworld Technology, Inc.) was used. Protein bands were subsequently detected using Super ECL Plus Western Blotting Substrate (BioGround Biotechnology Co. Ltd., Chongqing, China) and were visualized using the ChemiDoc Touch Imaging System (Bio-Rad Laboratories, Inc.). The gray value assay was performed using Image Lab software. All samples were run on the same gel (with nonessential lanes removed).

#### Intracellular calcium ions were detected using flow cytometry.

The levels of free cytosolic Ca^2+^ were measured using the cell-permeable Ca^2+^-sensitive fluorescent dye Fluo-4 AM (Beyotime Biotechnology, Shanghai, China). Each experimental group of PMVECs was stretched for 4 h in Ca^2+^- and Mg^2+^-containing Hanks’ Balanced Salt Solution (HBSS; Solarbio Technology Co., Ltd., Beijing, China). PMVECs were pretreated with the extracellular calcium-ion chelating agent EGTA (2 mM; ApexBio Technology) for 1 h before 18% CS was performed. PMVECs were stretched for 4 h and then incubated with 5 µM Fluo-4 AM for 30 min at 37°C in the dark, and the cells were then washed. A vehicle control group was used to confirm equal loading of Fluo-4 AM. The control group was maintained in Ca^2+^- and Mg^2+^-containing HBSS without Fluo-4 AM. We measured Ca^2+^ levels after treatment with HC-067047 (300 nM), an antagonist of TRPV4, which was defined as minimum fluorescence intensity. The fluorescence intensities of the other groups are expressed as %min. The fluorescence intensity of the Fluo-4 AM probe was analyzed by using a CytoFLEX (Beckman Coulter, Inc.) flow cytometer, and the results were analyzed using CytExpert software.

#### Statistical analysis.

The results are expressed as means ± SE. Results are representative of three independent experiments in quadruplicate samples. Comparisons between two groups were analyzed by the Student’s *t*-test. The statistical significance of the differences between various interventions was measured by either the one-way analysis of variance (ANOVA) followed by Tukey’s post-hoc test or two-way ANOVA. SPSS 21.0 software was used in this study. Statistical significance was indicated at *P* < 0.05.

## RESULTS

### 

#### High-fat diet attenuates VILI in mice.

**Fig. 1.**
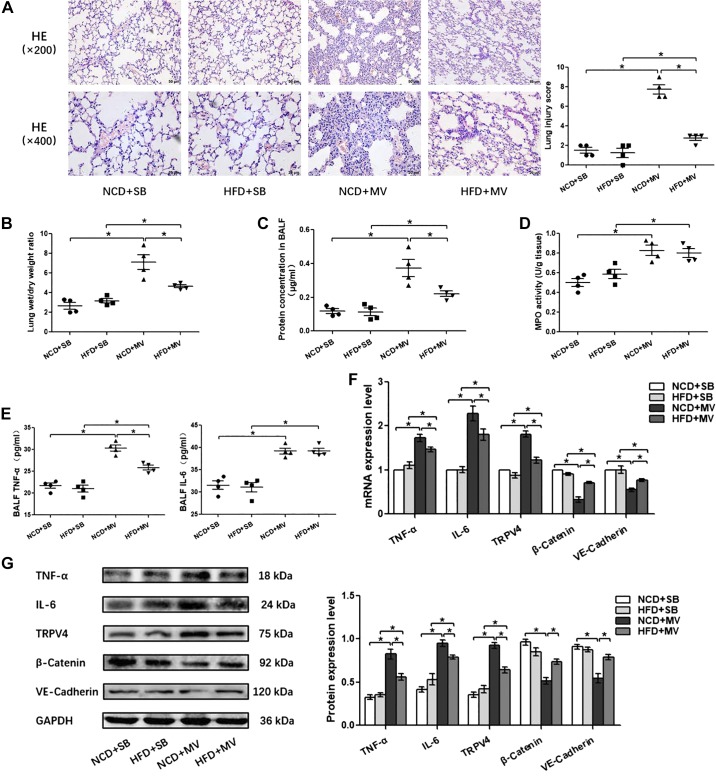
High-fat diet attenuates ventilator-induced lung injury (VILI) in mice. *A*: H&E staining (magnification, ×200 and ×400) and lung injury scores were used for the semiquantitative analysis of lung histopathological damage. *B*: wet/dry (W/D) weight ratio. *C*: protein concentrations in bronchoalveolar lavage fluid (BALF). *D*: myeloperoxidase (MPO) activity in lung tissue. *E*: proinflammatory cytokines TNF-α and IL-6 in BALF. *F*: quantitative real-time PCR (qRT-PCR) analysis shows the gene expression of TNF-α, IL-6, transient receptor potential vanilloid 4 (TRPV4), β-catenin, and VE-cadherin normalized to the gene expression of GAPDH. *G*: Western blot (WB) analysis shows the protein expression of TNF-α, IL-6, TRPV4, β-catenin, and VE-cadherin. The relative values are normalized to GAPDH; *n* = 4 mice from each group assayed in triplicate. The results are expressed as means ± SE. *P < 0.05. HFD, hight-fat diet; MV, mechanical ventilation; NCD, normal chow diet; SB, spontaneously breathing.

In the initial experiments, we investigated whether there was a difference in the response to VILI in HFD mice compared with NCD mice and whether the difference was favorable or harmful. Compared with the SB group, MV induced obvious inflammatory cell infiltration, edema in the lung, and thickening of the alveolar septa. These pathological changes were attenuated in HFD + MV mice compared with NCD + MV mice (Fig. 1*A*). The W/D weight ratio and protein concentrations in BALF were used to estimate the volume of pulmonary fluid and pulmonary microvascular endothelial barrier function. Significant increases in pulmonary leakage were observed in the MV group compared with that of the SB group, whereas pulmonary leakage was significantly decreased in HFD + MV mice compared with that in NCD + MV mice (Fig. 1, *B* and *C*). The MPO in lung tissue reflects neutrophil activity. The MPO in the MV group was elevated compared with that in the SB group; nevertheless, the MPO in the HFD + MV group showed no significant change compared with that in the NCD + MV group (Fig. 1*D*). Moreover, the levels of proinflammatory cytokines TNF-α and IL-6 in BALF were markedly increased after MV administration, and the TNF-α level in the HFD + MV group was significantly lower than that in the NCD + MV group (Fig. 1*E*). The expression of TNF-α and IL-6, mechanically sensitive calcium channel TRPV4, and adherens junctions β-catenin and VE-cadherin were analyzed in the lung. The mRNA levels of TNF-α, IL-6, and TRPV4 were significantly higher after MV administration, and the expression of all the above genes in the HFD + MV group was significantly lower than that in the NCD + MV group. The mRNA expression levels of β-catenin and VE-cadherin were significantly reduced by MV but significantly increased after MV administration in mice fed an HFD compared with those fed an NCD (Fig. 1*F*). The protein changes in TNF-α, IL-6, and TRPV4 were consistent with the changes in gene expression (Fig. 1*G*). Collectively, after mechanical ventilation for 4 h, the injury of the pulmonary microvascular barrier, the pulmonary inflammatory response, and the expression of TRPV4 were alleviated in HFD-induced obese mice compared with that in NCD normal mice, leading us to further explore the impact of obesity on VILI and its potential mechanisms.

#### Identification and morphological characterization of S-Exo, AT-Exo, and ADSC-Exo.

**Fig. 2.**
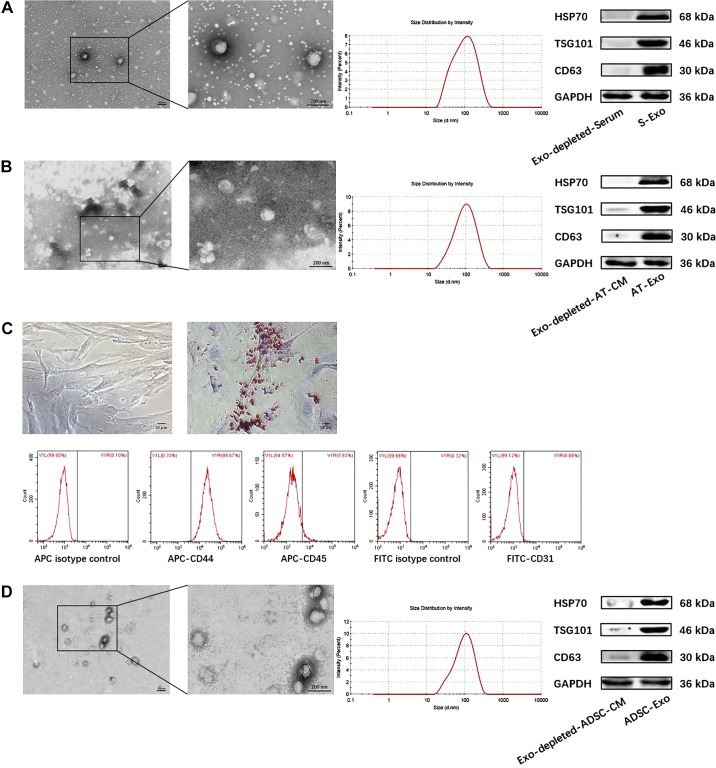
Identification and morphological characterization of serum exosome (S-Exo), adipose tissue exosome (AT-Exo), and adipose-derived stem cell exosome (ADSC-Exo). *A*, *B*, and *D*: (*left* to *right*): the morphology of exosomes is analyzed using transmission electron microscope (TEM), the size distribution of exosomes is analyzed using nanoparticle tracking analysis (NTA), and the exosomal markers heat shock protein 70 (HSP70), TSG101, and CD63 were analyzed using Western blotting (WB). *C*: characterization of ADSCs, including morphology; Oil Red O staining of adipogenic differentiation of ADSCs; and flow cytometry detection of ADSC immunophenotype. *n* = 4 samples from each group assayed in triplicate. All results are expressed as means ± SE.

The confirmation of the successful isolation of exosomes from the serum of HFD mice, adipose tissue, and ADSCs was performed using well-established methods such as TEM, NTA, and Western blotting (WB). We extracted the primary ADSCs, which exhibited a homogeneous population of spindle fibroblast-like cells. Positive Oil Red O staining (positive rate 71.25 ± 2.39%) was observed after the adipogenic induction of ADSCs for 14 days. The flow cytometry (FCM) results revealed that the ADSCs highly expressed the mesenchymal-derived cell marker CD44 (positive rate 98.04 ± 0.61%), whereas they lacked the blood-derived cell marker CD45 (positive rate 5.56 ± 0.38%) and the endothelial cell marker CD31 (positive rate 0.94 ± 0.14%), which was consistent with the phenotypic characteristics of ADSCs (Fig. 2*C*). S-Exo, AT-Exo, and ADSC-Exo were observed using TEM, and the results showed that these three exosomes, with typical sizes of 30–150 nm, exhibited typical cup-shaped morphology. In addition, using NTA to assess particle size, the average diameters of S-Exo, AT-Exo, and ADSC-Exo were 99.75 ± 2.93 nm, 104.9 ± 3.42 nm, and 96.75 ± 2.34 nm, respectively, which is consistent with the expected size of the exosomes. Furthermore, the exosomes from all three sources expressed the exosomal marker proteins HSP70, TSG101, and CD63 (Fig. 2, *A*, *B*, and *D*). These findings confirmed the efficiency of exosomes isolation from serum, adipose tissue, and ADSCs for subsequent experiments.

#### Uptake of S-Exo, AT-Exo, and ADSC-Exo by lung and PMVECs and the optimization of the optimal exosome intervention concentrations in vivo and in vitro.

**Fig. 3.**
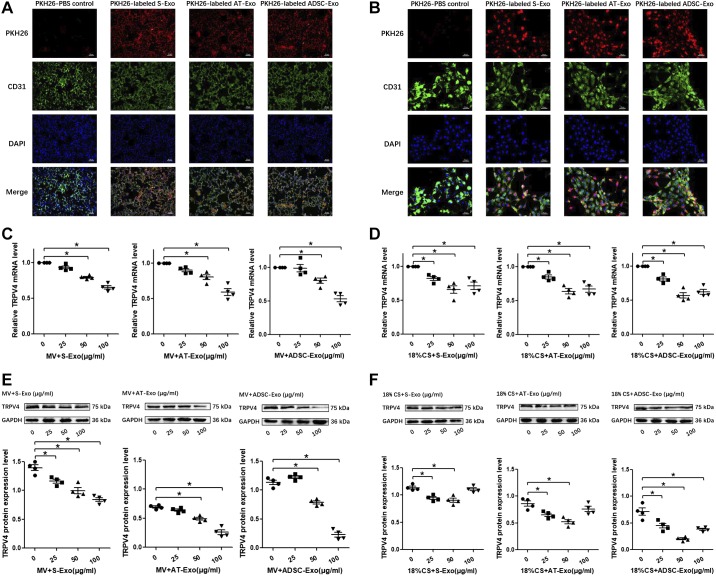
Uptake of serum exosome (S-Exo), adipose tissue exosome (AT-Exo), and adipose-derived stem cell exosome (ADSC-Exo) by lung tissue and pulmonary microvascular endothelial cells (PMVECs) and the optimization of the optimal exosome intervention concentrations in vivo and in vitro. *A*: uptake of S-Exo, AT-Exo, and ADSC-Exo by lung tissue. *B*: uptake of S-Exo, AT-Exo, and ADSC-Exo by PMVECs. In the fluorescence microscopy pictures, FITC-labeled CD31 was used to label the vascular endothelial cells (green), PKH26 was used to label the exosomes (red), and DAPI was used to detect the nucleus (blue). *C* and *E*: effects of three exosomes (S-Exo, AT-Exo, and ADSC-Exo) at different concentrations (0, 25, 50, and 100 μg/mL) on the expression of transient receptor potential vanilloid 4 (TRPV4) under mechanical ventilation in mice. The mRNA level of TRPV4 was evaluated by quantitative real-time PCR (qRT-PCR) and the protein level of TRPV4 was evaluated by Western blotting (WB). *D* and *F*: effects of three exosomes (S-Exo, AT-Exo, and ADSC-Exo) at different concentrations (0, 25, 50, and 100 μg/mL) on the expression of TRPV4 under 18% cyclic stretching in PMVECs. The mRNA level of TRPV4 was evaluated by qRT-PCR and the protein level of TRPV4 was evaluated by WB. *n* = 4 samples from each group assayed in triplicate. All results are expressed as means ± SE. *P < 0.05. MV, mechanical ventilation; CS, cyclic stretching.

To determine whether S-Exo, AT-Exo, and ADSC-Exo can be taken up by lung tissue and PMVECs, the three types of exosomes were labeled with PKH26 dye (red fluorescence) and administered to wild-type mice intratracheally in vivo and incubated with PMVECs in vitro. In the experimental groups, red fluorescence was observed in the lung tissue (Fig. 3*A*) and the cytoplasm of cells (Fig. 3*B*), indicating that the three types of exosomes were efficiently taken up by the lung tissue and PMVECs. In lung tissue, the positive rates of PKH26-labeled exosomes (S-Exo, AT-Exo, and ADSC-Exo) entering the pulmonary microvessels were 70.00 ± 2.04%, 76.25 ± 2.39%, and 71.25 ± 2.39%, respectively. In PMVECs, the positive rates of the PKH26-labeled three exosomes (S-Exo, AT-Exo, and ADSC-Exo) entering the endothelial cells were 88.75 ± 3.15%, 83.75 ± 1.25%, and 92.5 ± 1.44%, respectively. To examine the effects of S-Exo, AT-Exo, and ADSC-Exo on the key molecule TRPV4, the three types of exosomes at different concentrations (0, 25, 50, and 100 μg/mL) were intravenously administered 1 h before MV. The administration of S-Exo, AT-Exo, and ADSC-Exo reduced the mRNA and protein expression levels of TRPV4 in the lung in a dose-dependent manner at doses of 50 and 100 μg/mL. TRPV4 expression levels were lowest in the lung treated with 100 μg/mL S-Exo, AT-Exo, and ADSC-Exo. Thus, we treated the mice with 100 μg/mL exosomes in the subsequent MV experiments (Fig. 3, *C* and *E*). Then, we added S-Exo, AT-Exo, and ADSC-Exo at different concentrations (0, 25, 50, and 100 μg/mL) 1 h before 18% CS. We found that the mRNA and protein expression levels of TRPV4 in PMVECs were reduced, and a higher dose of exosomes (100 μg/mL) did not produce any increased effects compared with a dose of 50 μg/mL. The TRPV4 expression levels were lowest in the PMVECs that were treated with 50 μg/mL S-Exo, AT-Exo, and ADSC-Exo. Thus, we treated PMVECs with 50 μg/mL exosomes in the subsequent 18% CS experiments (Fig. 3, *D* and *F*).

#### The effects of S-Exo, AT-Exo, and ADSC-Exo on VILI in vivo.

**Fig. 4.**
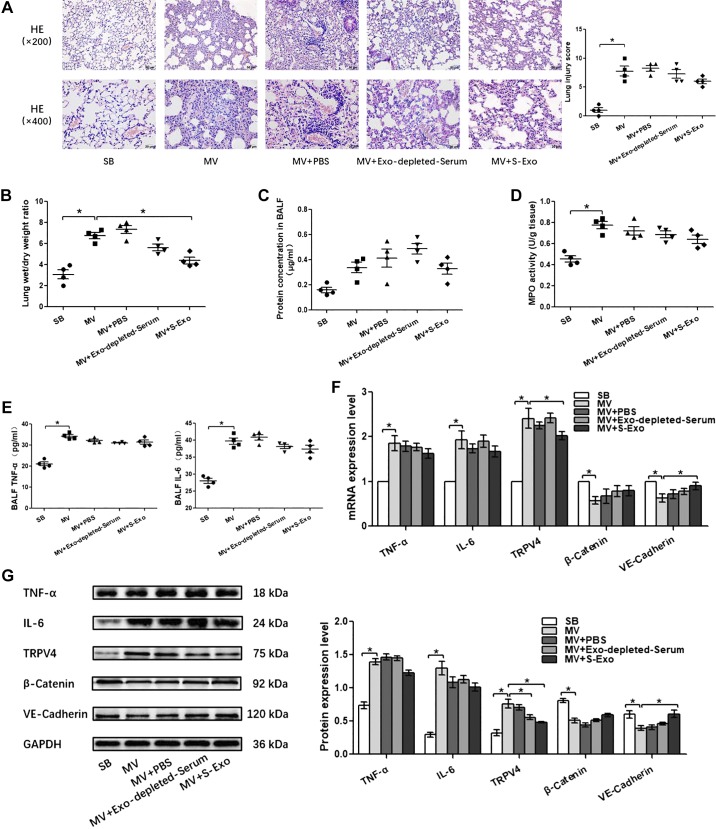
Exploring the role of serum exosome (S-Exo) in ventilator-induced lung injury (VILI) in vivo. *A*: H&E staining (magnification, ×200 and ×400), lung injury scores are used for the semiquantitative analysis of lung histopathologic damage. *B*: The wet/dry (W/D) weight ratio. *C*: protein concentrations in bronchoalveolar lavage fluid (BALF). *D*: myeloperoxidase (MPO) activity in lung tissue. *E*: proinflammatory cytokines TNF-α and IL-6 in BALF. *F*: quantitative real-time PCR (qRT-PCR) analysis shows the gene expression of TNF-α, IL-6, transient receptor potential vanilloid 4 (TRPV4), β-catenin, and VE-cadherin normalized to the gene expression of GAPDH. *G*: Western blot (WB) analysis shows the protein expression of TNF-α, IL-6, TRPV4, β-catenin, and VE-cadherin, and the relative values normalized to GAPDH. *n* = 4 mice from each group assayed in triplicate. The results are expressed as means ± SE. *P < 0.05. MV, mechanical ventilation; SB, spontaneously breathing.

**Fig. 5.**
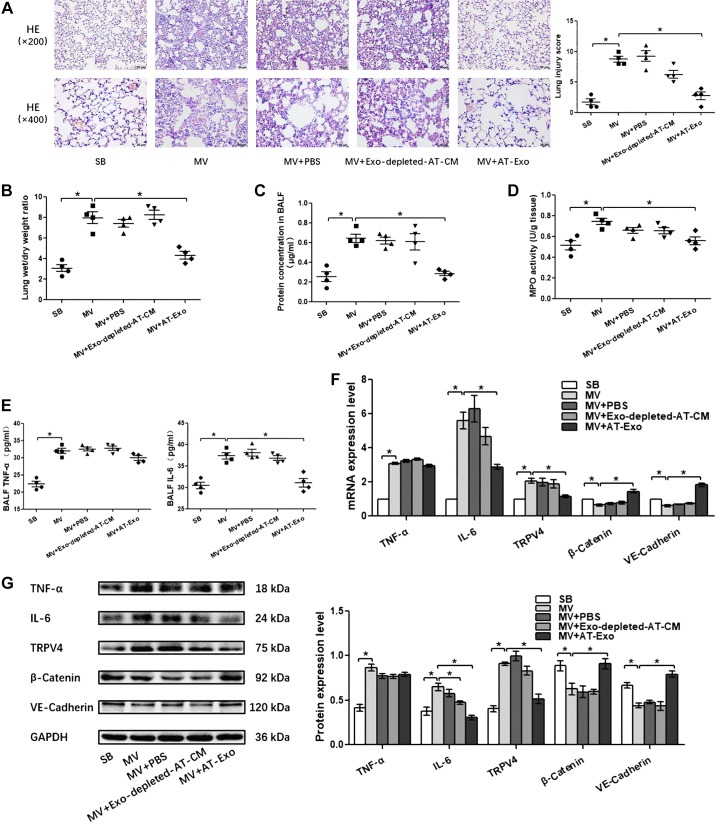
The effect of adipose tissue exosome (AT-Exo) on ventilator-induced lung injury (VILI) in vivo. *A*: hematoxylin and eosin (HE) staining (magnification, ×200 and ×400); lung injury scores are used for the semiquantitative analysis of lung histopathological damage. *B*: the wet/dry (W/D) weight ratio. *C*: protein concentrations in bronchoalveolar lavage fluid (BALF). *D*: myeloperoxidase (MPO) activity in lung tissue. *E*: proinflammatory cytokines TNF-α and IL-6 in BALF. *F*: quantitative real-time PCR (qRT-PCR) analysis shows the gene expression of TNF-α, IL-6, transient receptor potential vanilloid 4 (TRPV4), β-catenin, and VE-cadherin normalized to the gene expression of GAPDH. *G*: Western blot (WB) analysis shows the protein expression of TNF-α, IL-6, TRPV4, β-catenin, and VE-cadherin, and the relative values are normalized to GAPDH; *n* = 4 mice from each group assayed in triplicate. The results are expressed as means ± SE. *P < 0.05. CM, conditioned media; MV, mechanical ventilation; SB, spontaneously breathing.

**Fig. 6.**
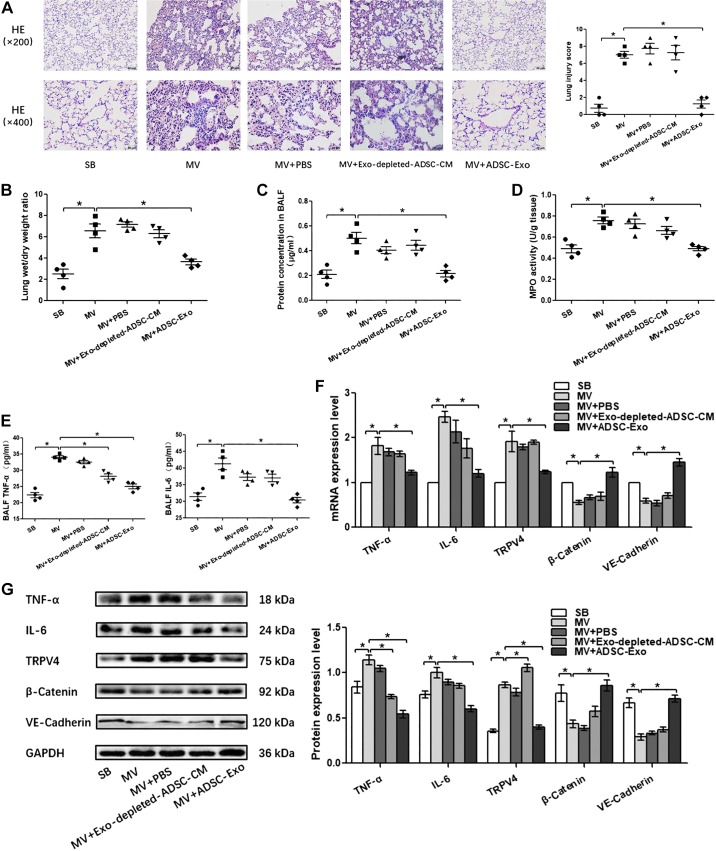
The effect of adipose-derived stem cell exosome (ADSC-Exo)-based treatment on ventilator-induced lung injury (VILI) in vivo. *A*: H&E staining (magnification, ×200 and ×400); lung injury scores are used for the semiquantitative analysis of lung histopathological damage. *B*: the wet/dry (W/D) weight ratio. *C*: protein concentrations in bronchoalveolar lavage fluid (BALF). *D*: myeloperoxidase (MPO) activity in lung tissue. *E*: proinflammatory cytokines TNF-α and IL-6 in BALF. *F*: quantitative real-time PCR (qRT-PCR) analysis shows the gene expression of TNF-α, IL-6, TRPV4, β-catenin, and VE-cadherin normalized to the gene expression of GAPDH. *G*: Western blot (WB) analysis shows the protein expression of TNF-α, IL-6, TRPV4, β-catenin, and VE-cadherin, and the relative values are normalized to GAPDH. *n* = 4 mice from each group assayed in triplicate. The results are expressed as means ± SE. *P < 0.05. MV, mechanical ventilation; SB, spontaneously breathing; ADSC-CM, ADSC conditioned media.

Pulmonary capillary endothelial barrier is a crucial target for high-stress mechanical ventilation, leading to endothelial hyperpermeability in VILI. Therefore, to further address the effects of adipose-derived exosomes on the homeostasis of pulmonary microvascular endothelial barrier in vivo, histopathological injury, W/D ratios, protein concentration in BALF, proinflammatory cytokine level, MPO activity, and the expression of β-catenin, VE-cadherin, and TRPV4 in lung tissue were assessed. Wild-type lean mice developed deteriorations in histological pulmonary edema and exacerbations in capillary leakage after high-stress mechanical ventilation, as manifested by increases in BALF protein concentrations and W/D ratios and decreases in adherens junction proteins β-catenin and VE-cadherin. At the same time, the inflammatory response was enhanced after high-stress mechanical ventilation, as evidenced by increased MPO activity in the lung tissue and increased proinflammatory factors TNF-α and IL-6 in the lung tissue or BALF. In addition, these changes were accompanied with the activation of TRPV4 channels. (Figs. 4, 5, and 6). S-Exo alleviated the W/D ratio (Fig. 4*B*), reduced the mRNA and protein expression level of TRPV4 (Fig. 4, *F* and *G*), and enhanced the mRNA and protein expression level of adherens junction proteins VE-cadherin (Fig. 4, *F* and *G*) in the lung tissue of VILI mice. However, other indicators related to the pulmonary microvascular endothelial barrier and inflammation did not change significantly (Fig. 4, *A*, *C*, *D*, *E*, *F*, and *G*). We further investigated the effect of AT-Exo and ADSC-Exo on VILI, and similarly, AT-Exo and ADSC-Exo significantly inhibited the mRNA and protein expression of TRPV4 (Fig. 5, *F* and *G*, and 6, *F* and *G*) and significantly attenuated pulmonary microvascular hyperpermeability in VILI mice, as evidenced by decreases in the lung injury score (Fig. 5*A* and 6*A*), W/D ratio (Fig. 5*B* and 6*B*), protein concentration in BALF (Fig. 5*C* and 6*C*), and increases in β-catenin and VE-cadherin in lung tissue (Fig. 5*F*, *G* and 6*F*, *G*). Regarding the inflammatory response, AT-Exo and ADSC-Exo can significantly inhibit MPO activity (Fig. 5*D* and 6*D*), mRNA, and protein levels of IL-6 in lung tissue (Fig. 5*F*, *G* and 6*F*, *G*) and reduce the expression of IL-6 in BALF (Fig. 5*E* and 6*E*). In addition, ADSC-Exo can significantly inhibit the mRNA and protein levels of TNF-α in lung tissue (Fig. 6, *F* and *G*) and the expression of TNF-α in BALF (Fig. 6*E*). Collectively, all three adipose-derived exosomes had protective effects on VILI, among which ADSC-Exo was the most significant.

#### S-Exo, AT-Exo, and ADSC-Exo partly suppresses PMVEC inflammation and promote PMVEC barrier integrity after 18% CS insult through inhibition of TRPV4/Ca^2+^ signaling in vitro.

**Fig. 7.**
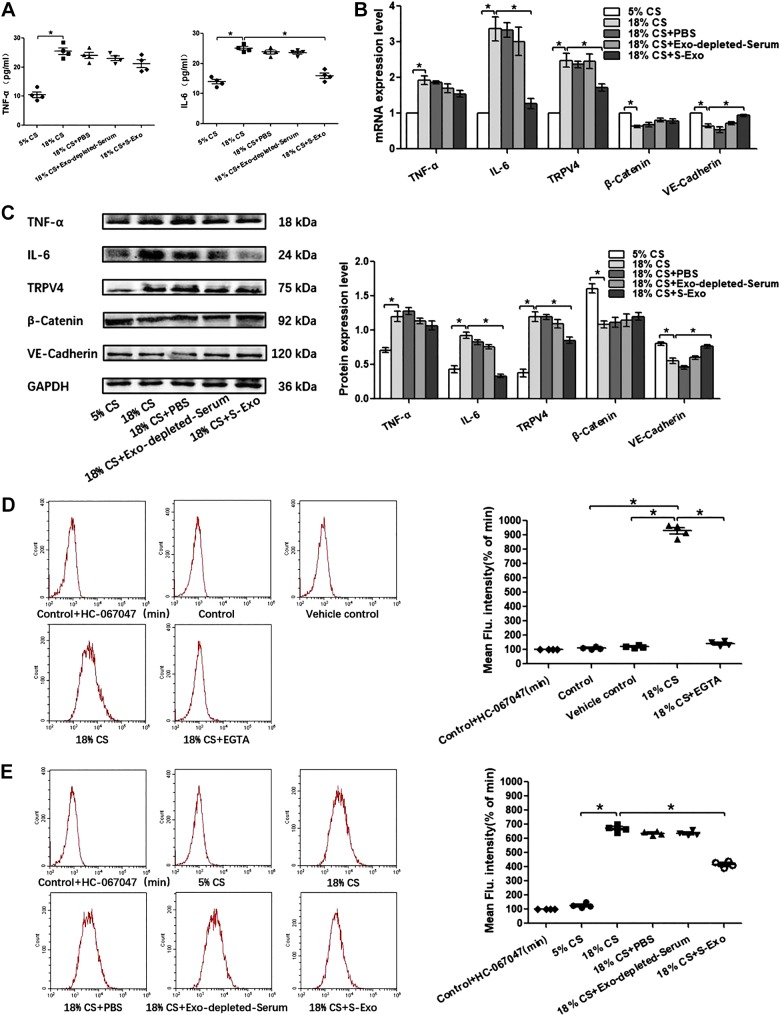
Serum exosome (S-Exo) partly suppresses pulmonary microvascular endothelial cell (PMVEC) inflammation and promotes barrier function after 18% cyclic stretching (CS) through inhibiting transient receptor potential vanilloid 4 (TRPV4)/Ca^2+^ signaling in vitro. *A*: proinflammatory cytokines TNF-α and IL-6 in cell culture supernatant. *B*: quantitative real-time PCR (qRT-PCR) analysis shows the gene expression of TNF-α, IL-6, TRPV4, β-catenin, and VE-cadherin normalized to the gene expression of GAPDH. *C*: Western blot (WB) analysis shows the protein expression of TNF-α, IL-6, TRPV4, β-catenin, and VE-cadherin, and the relative values are normalized to GAPDH. *D* and *E*: intracellular calcium ions are detected by flow cytometry (FCM). *n* = 4 cultures from each group assayed in triplicate. The results are expressed as means ± SE. *P < 0.05.

**Fig. 8.**
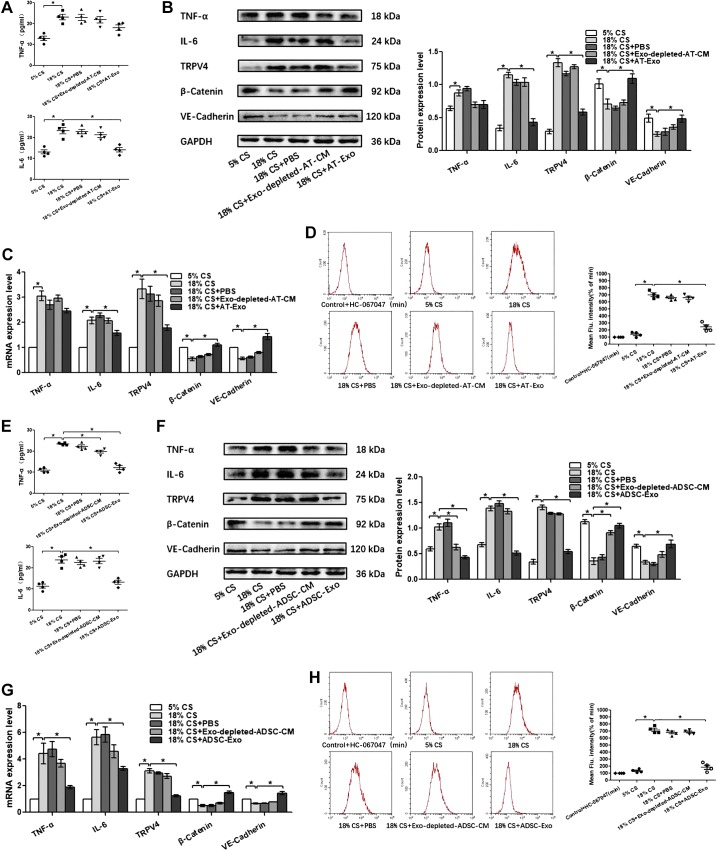
Adipose tissue exosome (AT-Exo) and adipose-derived stem cell exosome (ADSC-Exo) promote pulmonary microvascular endothelial cell (PMVEC) barrier function and suppress inflammation after 18% cyclic stretching (CS) through inhibiting transient receptor potential vanilloid 4 (TRPV4)/Ca^2+^ signaling in vitro. *A* and *E*: proinflammatory cytokines TNF-α and IL-6 in cell culture supernatant. *B* and *F*: Western blot (WB) analysis shows the protein expression of TNF-α, IL-6, TRPV4, β-catenin, and VE-cadherin, and the relative values are normalized to GAPDH. *C* and *G*: quantitative real-time PCR (qRT-PCR) analysis shows the gene expression of TNF-α, IL-6, TRPV4, β-catenin, and VE-cadherin normalized to the gene expression of GAPDH. *D* and *H*: intracellular calcium ions are detected by flow cytometry (FCM); *n* = 4 cultures from each group assayed in triplicate. The results are expressed as means ± SE. *P < 0.05. ADSC-CM, ADSC conditioned media.

Given the marked beneficial effects of adipose-derived exosomes on the pulmonary endothelial barrier dysfunction in vivo, PMVECs were subjected to 18% cyclic stretching (CS) to imitate pathological high tidal volume mechanical ventilation in vitro. To explore the therapeutic effects of S-Exo, AT-Exo, and ADSC-Exo on 18% CS insult and the potential mechanism, proinflammatory cytokine level, the expression of β-catenin, VE-cadherin, and TRPV4 and intracellular calcium ions were assessed. We found that S-Exo reversed 18% CS-induced increases of IL-6 in cell culture supernatants, whereas TNF-α did not change significantly (Fig. 7*A*). The upregulated mRNA and protein expression levels of IL-6 and TRPV4 were obviously reduced, whereas downregulated mRNA and protein expression of VE-cadherin was obviously increased in the 18% CS + S-Exo group compared with those in the 18% CS group (Fig. 7, *B* and *C*). AT-Exo decreased the level of IL-6 in cell culture supernatants, with no changes in TNF-α level (Fig. 8*A*). The mRNA and protein expression levels of β-catenin and VE-cadherin were significantly elevated, whereas the mRNA and protein expression levels of IL-6 and TRPV4 were significantly decreased by the treatment of AT-Exo in PMVECs under 18% CS challenge (Fig. 8, *B* and *C*). It is worth noting that ADSC-Exo can simultaneously reduce the 18% CS-induced increase in TNF-α and IL-6 in cell culture supernatants (Fig. 8*E*). Enhanced mRNA and protein expression of TNF-α, IL-6, TRPV4, and decreased mRNA and protein expression of β-catenin and VE-cadherin in PMVECs induced by 18% CS were significantly inhibited by ADSC-Exo (Fig. 8, *F* and *G*). Thus, three adipose-derived exosomes could significantly restore endothelial barrier integrity and inhibit the inflammatory response in PMVECs under 18% CS insult, accompanied by suppressing TRPV4 activation. As a calcium-permeable channel, TRPV4 is a key protein in sensing mechanical stress in endothelial cells; therefore, we further determined whether TRPV4’s activation is related to intracellular Ca^2+^ changes. As shown in Fig. 7*D*, after 18% CS, the levels of intracellular calcium ions were significantly increased compared with that in the control group. After the extracellular calcium ion chelator EGTA was administered, intracellular calcium ion levels were significantly decreased. There were no significant differences among the control group, vehicle control group, or 18% CS + EGTA group, indicating that 18% CS mainly caused extracellular calcium-ion influx, which most likely occurred through the TRPV4 channel (Fig. 7*D*). Furthermore, the intracellular calcium ion concentration increased significantly due to 18% CS insult. Intervention with S-Exo, AT-Exo, and ADSC-Exo significantly decreased intracellular calcium ion concentration. This trend is basically consistent with alternations in TRPV4 activity (Fig. 7*E* and 8, *D* and *H*). Collectively, these findings suggest that the therapeutic effects of S-Exo, AT-Exo, and ADSC-Exo on 18% CS are mediated at least in part by inhibiting TRPV4/Ca^2+^ pathway.

#### TRPV4/Ca^2+^ signaling pathway mediates ADSC-Exo’s protection on pulmonary endothelial barrier in VILI.

**Fig. 9.**
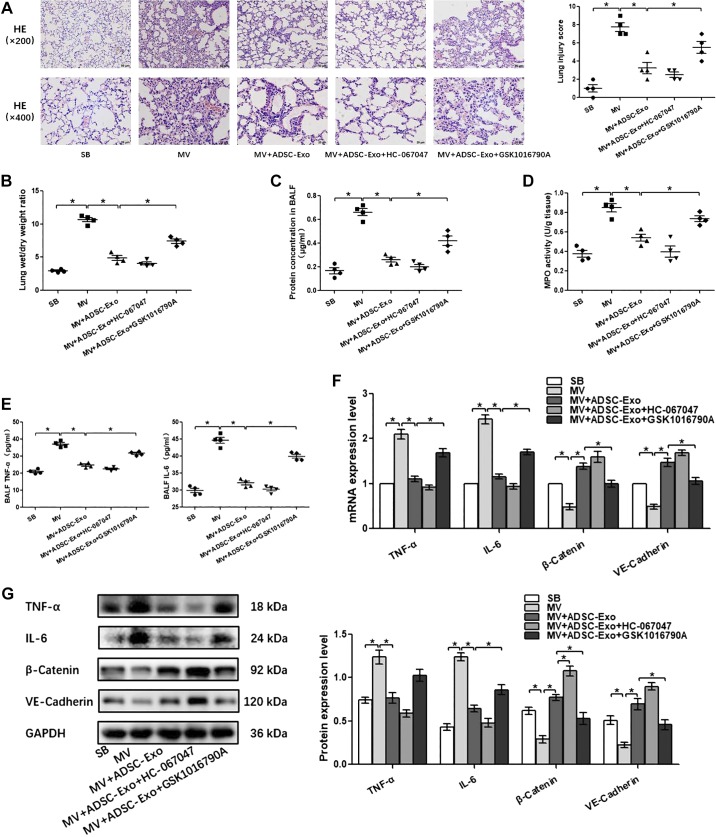
Effects of the combination of adipose-derived stem cell exosome (ADSC-Exo) and transient receptor potential vanilloid 4 (TRPV4) agonist (GSK1016790A)/antagonist (HC-067047) on ventilator-induced lung injury (VILI) in vivo. *A*: H&E staining (magnification, ×200 and ×400); lung injury scores were used for the semiquantitative analysis of lung histopathological damage. *B*: the wet/dry (W/D) weight ratio. *C*: protein concentrations in bronchoalveolar lavage fluid (BALF). *D*: myeloperoxidase (MPO) activity in lung tissue. *E*: proinflammatory cytokines TNF-α and IL-6 in BALF. *F*: quantitative real-time PCR (qRT-PCR) analysis shows the gene expression of TNF-α, IL-6, β-catenin, and VE-cadherin normalized to the gene expression of GAPDH. *G*: Western blot (WB) analysis shows the protein expression of TNF-α, IL-6, β-catenin, and VE-cadherin, and the relative values are normalized to GAPDH. *n* = 4 mice from each group assayed in triplicate. The results are expressed as means ± SE. *P < 0.05. MV, mechanical ventilation; SB, spontaneously breathing.

**Fig. 10.**
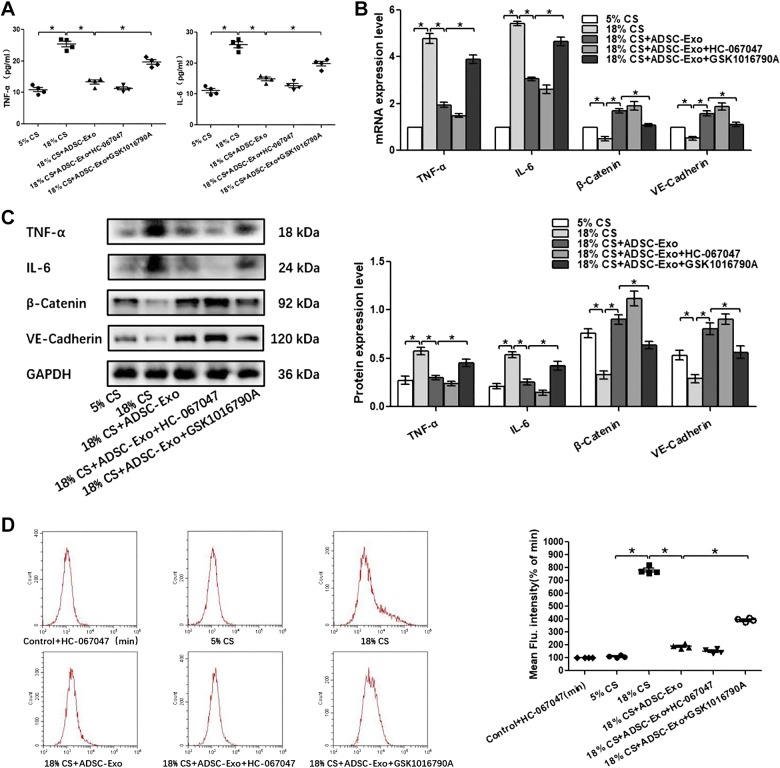
Effects of the combination of adipose-derived stem cell exosome (ADSC-Exo) and transient receptor potential vanilloid 4 (TRPV4) agonist (GSK1016790A)/antagonist (HC-067047) on 18% cyclic stretching (CS) in vitro. *A*: proinflammatory cytokines TNF-α and IL-6 in cell culture supernatant. *B*: quantitative real-time PCR (qRT-PCR) analysis shows the gene expression of TNF-α, IL-6, β-catenin, and VE-cadherin normalized to the gene expression of GAPDH. *C*: Western blot (WB) analysis shows the protein expression of TNF-α, IL-6, β-catenin, and VE-cadherin, and the relative values are normalized to GAPDH. *D*: intracellular calcium ions are detected by flow cytometry (FCM). *n* = 4 cultures from each group assayed in triplicate. The results are expressed as means ± SE. *P < 0.05.

With the use of TRPV4 agonist (GSK1016790A) or TRPV4 antagonist (HC-067047), we further confirmed the involvement of the TRPV4/Ca^2+^ pathway in ADSC-Exo’s protection against VILI in vivo and in vitro. As shown in Figs. 9 and 10, we found that pretreatment with GSK1016790A partially reversed the protective effects of ADSC-Exo on lung injury in VILI. The suppressive effects of ADSC-Exo on VILI inflammation, such as the decreases in MPO activity and expression of TNF-α and IL-6, were significantly reversed by pre-treatment of GSK1016790A, whereas the improving effects of ADSC-Exo on VILI barrier function, such as the decreases in BALF protein concentration and W/D ratio and the increases in β-catenin and VE-cadherin expression, were significantly suppressed by pretreatment of GSK1016790A. The combination of HC-067047 with ADSC-Exo did not exert additional protection against VILI. These results suggest that ADSC-Exo can protect VILI barrier and inhibit inflammation directly via the suppression of TRPV4 channel.

#### One-shot treatment with ADSC-Exo alleviates pulmonary endothelial barrier hyperpermeability and inflammation response after VILI in vivo and in vitro.

**Fig. 11.**
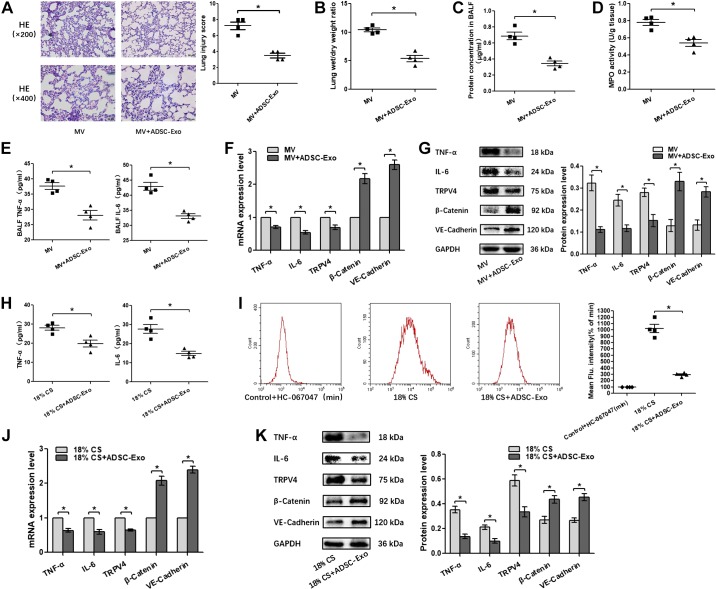
One-shot treatment with adipose-derived stem cell exosome (ADSC-Exo) alleviates pulmonary endothelial barrier hyperpermeability and inflammation response after ventilator-induced lung injury (VILI) in vivo and in vitro. *A*: H&E staining (magnification, ×200 and ×400); lung injury scores were used for the semiquantitative analysis of lung histopathological damage. *B*: the wet/dry (W/D) weight ratio. *C*: protein concentrations in bronchoalveolar lavage fluid (BALF). *D*: myeloperoxidase (MPO) activity in lung tissue. *E*: proinflammatory cytokines TNF-α and IL-6 in BALF. *F* and *J*: quantitative real-time PCR (qRT-PCR) analysis shows the gene expression of TNF-α, IL-6, transient receptor potential vanilloid 4 (TRPV4), β-catenin, and VE-cadherin normalized to the gene expression of GAPDH. *G* and *K*: Western blot (WB) analysis shows the protein expression of TNF-α, IL-6, TRPV4, β-catenin, and VE-cadherin, and the relative values are normalized to GAPDH. *H*: proinflammatory cytokines TNF-α and IL-6 in cell culture supernatant. *I*: intracellular calcium ions are detected by flow cytometry (FCM). *n* = 4 mice or cultures from each group assayed in triplicate. The results are expressed as means ± SE. *P < 0.05. MV, mechanical ventilation; CS, cyclic stretching.

To explore the therapeutic potential of ADSC-Exo, mice and PMVECs were administered with ADSC-Exo immediately after MV or 18% CS insult. The treatment of ADSC-Exo significantly attenuated pulmonary microvascular hyperpermeability in VILI mice, as evidenced by decreases in the lung injury score, W/D ratio, and protein concentration in BALF and increases in β-catenin and VE-cadherin. Moreover, ADSC-Exo can significantly inhibit MPO activity and the mRNA and protein levels of IL-6 and TNF-α in lung tissue as well as the expression of IL-6 and TNF-α in BALF (Fig. 11, *A*, *B*, *C*, *D*, *E*, *F*, and *G*). In PMVECs, ADSC-Exo also showed beneficial effects on restoring endothelial barrier and inhibiting inflammatory response. In addition, intracellular calcium ion concentration was also significantly reduced by ADSC-Exo administration in PMVECs, accompanied by TRPV4 inhibition (Fig. 11, *H*–*K*). Collectively, these findings suggest a therapeutic potential of ADSC-Exo for treating VILI at least in part by inhibiting the TRPV4/Ca^2+^ signaling pathway.

## DISCUSSION

Clinically, obesity is associated with high susceptibility to ARDS; however, the resultant mortality is low. A recent meta-analysis showed that obese patients with ARDS/ALI have a better survival advantage, consistent with the concept of the “obesity paradox” (59). Related studies found that mechanical ventilation as an important treatment and support means for ARDS patient is subject to the “obesity paradox” in severe patients receiving mechanical ventilation (58). Through in vivo and in vitro experiments, we investigated whether the protective effects of obesity on VILI could explain this phenomenon and its related mechanisms. In this study, mechanical ventilation at 30 mL/kg was used to induce VILI in vivo (37), and 18% cyclic stretching was used to simulate VILI in vitro (18). The results showed that HFD mice were less affected by mechanical ventilation and had a lower lung injury score, reduced permeability changes, and reduced inflammatory responses. We also found protective effects of adipose-derived exosomes on VILI, of which AT-Exo and ADSC-Exo were predominant.

Most studies on the protective effect of obesity on lung injury have focused on beneficial adipocytokines. For example, omentin (36), adiponectin, and secreted frizzled-related protein 5 (SFRP5) can effectively reduce inflammation (33, 47). Furthermore, secretory factors in adipose tissue extracts promote angiogenesis in vivo and in vitro (25, 38). In addition to secreting adipocytokines, adipose tissue also releases vesicles called exosomes. As a new means of intercellular communication, adipose tissue has attracted increasing attention and is involved in various physiological processes, such as cell metabolism, differentiation, and proliferation (56). Exosomes derived from adipose are mainly obtained from mice serum, adipose tissue explants, primary ADSCs in rats, and differentiated 3T3-L1 cell lines (11, 56). In this study, we focused on exosomes from the circulation, tissue, and stem cell sources.

Mechanical ventilation is an important means of support for ARDS patients; however, it may also cause VILI. Research has found that excessive lung ventilation can activate serum glucocorticoid-regulated kinase 1 (SGK1) to further activate the mechanically sensitive calcium channel TRPV4 (29), increase extracellular Ca^2+^ influx, and cause the contraction of endothelial cell borders to increase cell membrane permeability (12) and release proinflammatory cytokines. However, the inhibition of TRPV4 attenuates lung barrier permeability increase and proinflammatory cytokine release caused by high tidal volume ventilation, serving as a beneficial protection against mechanical ventilation (34). In human retinal microvascular endothelial cells, selective TRPV4 agonist activates nonselective cation currents, increases intracellular calcium, interrupts organization of endothelial F-actin, downregulates occluding expression, and reverses the remodeling of adhesion junctions (VE-cadherin and β-catenin), thus increasing the permeability of microvascular endothelial cell monolayers. Collectively, TRPV4 plays an important role in Ca^2+^ signaling, cytoskeletal remodeling, and barrier function (35).

We found that pulmonary capillary leakage was increased after mechanical ventilation induced VILI, as manifested by the increase in protein concentrations in BALF and W/D ratios as well as the exaggerated inflammatory responses in vivo. PMVEC hyperpermeability induced by 18% cyclic stretching was associated with the activation of TRPV4 and extracellular calcium influx in vitro. The protective effects of adipose-derived exosomes on mechanical ventilation and 18% cyclic stretching are improved mainly by the inhibition of TRPV4, the enhancement of lung barrier function, and the reduction in inflammation. Exo-depleted-serum, Exo-depleted-AT-CM, and Exo-depleted-ADSC-CM have almost no effect on VILI. Overall, S-Exo has weaker effects than AT-Exo and ADSC-Exo on the barrier protection and anti-inflammatory effects of VILI, whereas ADSC-Exo has the most protective effect on VILI. To explore whether adipose-derived exosomes’ protective effects on pulmonary endothelial barrier in VILI are mediated by inhibiting TRPV4 directly, we further investigated the effects of combined application of ADSC-Exo and TRPV4 agonist or antagonist on VILI and found that the protective effect of ADSC-Exo could be reversed after TRPV4 agonist administration. Therefore, we speculate that the inhibition of TRPV4 by ADSC-Exo or adipose-derived exosomes may be direct. Moreover, therapeutic treatment with ADSC-Exo was also effective in reinforcing the endothelial cell barrier and suppressing endothelial inflammation by inhibiting the TRPV4/Ca^2+^ pathway in vivo and in vitro.

Many researchers have found that exosomes secreted by stem cells play an active role in healing tissue damage (20), stimulating angiogenesis (14) and reducing inflammation (10). Because adipose tissue also contains a large number of adipose stem cells, we observed that adipose-derived ADSCs play an important role in the protective mechanism against VILI. Injury repair studies of the effects of adipose tissue on the healing of skin lesions, especially those that address the exosomes secreted by ADSCs, revealed that ADSC-Exo may act through the Wnt/β-catenin signaling pathway (28) or by targeting Toll-like receptor 4 (TLR4) to inhibit inflammatory cytokines (26). ADSC-Exo also promotes fibroblast proliferation and migration through the phosphatidylinositol 3-kinase (PI3K)/Akt signaling pathway (55). Our study reports that the protective effect of adipose-derived exosomes on VILI can be at least partially mediated by inhibiting mechanically sensitive calcium channel TRPV4 and extracellular calcium influx. Meanwhile, the inhibitory effects of AT-Exo and ADSC-Exo were found to be significantly greater than those of S-Exo. Moreover, S-Exo in the circulatory system is relatively complex in terms of its source and cargo substance (13, 43). Adipose tissue is an important organ that regulates metabolism and interactions with other organs by secreting various adipokines and hormones. Therefore, other adipokines may also be involved in this protection.

microRNAs (miRNAs) can be secreted into exosomes and serve as key regulators of molecules involved in regulating mRNA gene expression in most cell types. These exosomes containing miRNA can work locally or enter circulation and further be taken up into distant cells types to regulate gene expression and function of recipient cells in distal tissues (54). Exosomal microRNAs are even considered to be genetic adipokines (19). Studies have found that exosomes secreted by mesenchymal stem cells promote endothelial cell angiogenesis by transferring miRNA-125a (24). In addition, exosomes secreted by ADSCs have been reported to regulate angiogenesis in brain microvascular endothelial cells via miRNA-181b (53). However, small RNA sequencing revealed that miR-146a is significantly overexpressed and has high abundance in multiple types of stem cells (21, 44). miR-146a is involved in the repairment of injury and inhibition of inflammation (44, 52, 60). Related studies show that miR-146a can reduce the expression of proinflammatory cytokines TNF-α and IL-6 in VILI mice by inhibiting the Toll-like receptor 4 (TLR4) signaling pathway (6, 9, 22), which plays an important role in high-tidal volume mechanical ventilation (8). Although the regulatory interaction between TLR4 and TRPV4 signaling cascades in endothelial cells remains unexplored, TLR4-mediated effects in other cell types do require a Ca^2+^ entry pathway through other TRP channel family members (7). Therefore, we speculated that adipose-derived exosomes might promote pulmonary injury repairment and alleviate inflammatory response via exosomal miRNAs.

Currently, two clinical applications have been developed for mesenchymal stem cells in the treatment of patients with severe ARDS, which demonstrate the ability of mesenchymal stem cells to ameliorate respiratory defects, dysregulated hemodynamics, and multiple organ failure (40), while cell-free exosomes could be used as superior pharmaceutical therapeutics, depending on their unique characteristics such as lack of cytotoxicity, high drug carrying capacity, and low immunogenicity to facilitate the transport of genetic material at nanoparticle size (4). However, the limitation of our study is that, as an initial experimental study, we focused on the observation of adipose-derived exosomes’ potential effects on VILI without fully exploring their fundamental mechanisms. In our ongoing research, we are profiling serum exosomal miRNA alternation in wild-type lean mice and high-fat obese mice after VILI challenge. Further experimental and clinical studies are still needed to elucidate the fundamental mechanisms that mediated protective effects of exosomes derived from the obese in the setting of VILI.

In conclusion, our study showed that HFD-induced obesity plays a protective role in VILI by suppressing the pulmonary endothelial barrier hyperpermeability and inflammatory response via adipose-derived exosomes, at least partially, through inhibiting the TRPV4/Ca^2+^ signaling pathway, further providing novel insights into the “obesity paradox” in VILI and suggesting potential exosome-based therapeutics for patients with VILI in clinical practice.

## GRANTS

This study was supported by the National Natural Science Foundation of China (Grant no. 81670071), the National Natural Science Foundation for Young Scholars of China (Grant no. 81800083), the China Postdoctoral Science Foundation (2019M653832XB), and Natural Science Foundation of Chongqing, China (Grant no. cstc2019jcyj-zdxmX0031).

## DISCLOSURES

No conflicts of interest, financial or otherwise, are declared by the authors.

## AUTHOR CONTRIBUTIONS

Q.Y., D.W., and D.Q. conceived and designed research; Q.Y., X.W., and X.T. performed experiments; Q.Y., D.W., X.W., X.T., and D.Q. analyzed data; Q.Y., D.W., X.T., and D.Q. interpreted results of experiments; Q.Y. prepared figures; Q.Y. drafted manuscript; Q.Y., D.Q., J.H., Y.Z., W.D., and T.Z. edited and revised manuscript; Q.Y., D.W., X.W., X.T., D.Q., J.H., Y.Z., W.D., and T.Z. approved final version of manuscript.
